# Evaluation of Patient-Reported Symptoms and Functioning after Treatment for Endometrial Cancer

**DOI:** 10.3390/curroncol29080414

**Published:** 2022-07-22

**Authors:** Monika Sobočan, Dorotea Gašpar, Estera Gjuras, Jure Knez

**Affiliations:** 1 Division of Gynecology and Perinatology, University Medical Centre Maribor, 2000 Maribor, Slovenia; jure.knez@ukc-mb.si; 2 Department of Obstetrics and Gynecology, Faculty of Medicine, University of Maribor, 2000 Maribor, Slovenia; dorotea.gaspar@sb-ms.si (D.G.); estera.gjuras@sb-celje.si (E.G.)

**Keywords:** patient-reported outcomes, endometrial cancer, sexual function, cancer treatment

## Abstract

The overall survival of women with endometrial cancer is excellent after management. Different management strategies are associated with variable patient-reported outcomes (PROs). Evaluating PROs in the follow-up period can aid in better counseling and intervention for PRO improvement. This study aimed to evaluate the properties of the Slovenian translation of the EORTC QLQ-EN24 assessment scale. Women treated at the University Medical Centre Maribor, Slovenia, between January 2016 and December 2019 were invited to report their symptoms using the EORTC QLQ-EN24 questionnaire. Data were correlated with treatment modalities and clinical characteristics. The median age of participants in our study was 61 years old. PROs were not specific to therapy or clinical characteristics. Overall, women who more frequently reported gastrointestinal symptoms, muscular pain, or back pain also had statistically more frequently decreased levels of PROs in other assessed areas. Women who reported sexual or vaginal problems more often reported significantly poorer body images. Sexual activity within 4 weeks prior to completing the scale was reported by 39% of women. Sexual functioning assessments also showed important correlations between sexual interest, enjoyment, and activity. Individualized follow-ups addressing PROs should be offered to better address concerns and improve long-term outcomes in women with endometrial cancer.

## 1. Introduction

Endometrial cancer is the most common gynecological malignancy. The 5-year overall survival after treatment is excellent and exceeds 90% in the early stages [1]. Hence, quality of life (QOL) after management is especially important in this group of women. Even though early detection and multimodal treatments decrease overall cancer deaths and improve prognosis, cancer remains a chronic illness. An integral part of QOL assessment is the patient-reported outcome (PRO). PROs represent dimensions of social, financial, psychosocial, and physical activities, as reported subjectively by the patients themselves. PROs are always subjective interpretations of patients and their health assessments may vary (from the assessments of health professionals or relatives) [2,3,4].

Few studies have investigated PROs in endometrial cancer [5]. A recent systematic review showed that PROs are assessed with a heterogeneous selection of tools, such as the European Organization for Research and Treatment of Cancer Quality of Life Questionnaire-Core 30 (EORTC QLQ-C30), the Short Form 36 Questionnaire (SF-36), the Functional Assessment of Cancer Therapy-General (FACT-G), and the Female Sexual Function Index (FSFI) [6].

Studies have reported on the major categories of factors impacting patient-related outcomes, including body mass index (BMI) and/or physical activity, treatment, and sexual functions. Evidence about other, less-studied categories, which might be important in patient-related outcomes, is also emerging [6].

Obesity is related to lower QOL and physical functioning after EC management [6]. This is likely due to the reduced physical functioning and lower activity levels also reported by obese EC patients compared to their normal weight counterparts [7,8]. This observation is particularly important for EC patients, who are less likely to meet recommended activity and BMI guidelines compared to women without EC diagnoses [9].

The next major factor influencing PROs is the treatment of EC. Studies have reported that patients treated with minimally invasive surgery (compared to laparotomies) have more favorable outcomes in general health, physical functioning, and pain [10,11,12]. A meta-analysis showed that overall QOL outcomes are better if women are treated by laparoscopies than laparotomies [6]. Considering adjuvant therapy, vaginal brachytherapy is associated with better PROs in comparison to external beam radiation [13,14].

The third aspect impacting PROs is sexual functioning. Two cross-sectional studies, both of which surveyed patients within five years of EC diagnoses, reported conflicting findings related to aging and sexual function. A study conducted on Chinese EC survivors concluded that sexual dysfunctions were more common in older women [15]. However, a recent Australian study found that older women were more likely to report better sexual well-being compared to younger women [16]. These studies were performed in different populations and included relatively low numbers of women. Differences in the reported outcomes and factors influencing these outcomes support further assessments of the different factors influencing reduced sexual functioning after EC management.

Factors such as emotional distress, diagnostic delay, menopausal symptoms, and sleep quality, although less commonly examined, were also important characteristics in the EC patient experiences in a recent systematic review [6]. Active coping (as opposed to passive coping) was related to lower mortality, suggesting that counseling (to increase the use of this strategy) could be important after EC diagnosis [17]. Further, Honerlaw et al., in their work on emotional distress, inflammatory cytokines, and pain following EC surgery, observed that changes in IL-6 levels were related to changes in pain, potentially providing an important biomarker to monitor pain and PRO levels during follow-up [18].

The heterogeneity of QOL assessment and PRO assessment in EC warrants further investigation into reported PROs in different EC patient subgroups. This study aimed to validate an endometrial cancer-specific PRO tool and assess the impact of therapy on PROs.

## 2. Patients, Instruments, and Methods

### 2.1. Patient Recruitment

Women treated for endometrial cancer at the University Medical Centre Maribor from January 2016 to December 2019 were identified from the institutional registry. Patients who were alive and had a known contact address (after initial identification from the registry) were invited to participate in this study. All women were invited by post to complete the EORTC QLQ-EN24 questionnaire and return it within a timeframe of two weeks to the University Medical Centre Maribor. This study was approved by an Institutional Review Board (IRB, reg no. UKC-MB-KME-22/21). Participants indicated their consent to participate in the study by returning the enclosed questionnaire (as indicated in the cover letter). The need for a written consent form was waived by the IRB.

### 2.2. Instrument Language Adaptation

The EORTC QLQ-EN24 instrument was developed specifically for endometrial cancer patients to evaluate the long-term quality of life-related outcomes. It was developed to incorporate functional scales on sexual interest, activity, enjoyment, and the Likert scale symptom items. The symptom items include the evaluation of lymphedema presence, urological and gastrointestinal symptoms, the assessment of body image, sexual problems, pain symptoms, tingling/numbness, hair loss, and taste changes [19]. The scale is available from the EORTC website: https://qol.eortc.org/questionnaires/ (accessed on 11 May 2022).

The EORTC quality of life questionnaire for endometrial cancer (English version of module EN24) was taken as a basis to create the Slovenian version [19]. The procedure was consistent with translation guidelines [20]. The EORTC translation office first presented the English original version. Two independent forward translations (from English to Slovenian) were performed. The first Slovenian version was created. Later, two independent backward translations (from Slovenian to English) were performed. Translation issues were discussed among researchers to reach a consensus. Afterward, the Slovenian version, together with the back translation into English, were presented to the EORTC translation office. After the translation, pilot testing was performed to identify and solve any potential problems in the translation, including vocabulary issues and expressions that would be most suitable for the target population. Pilot testing of the Slovenian version was conducted on 10 patients with endometrial cancer who attended follow-up appointments at the University Medical Centre Maribor. According to the patients’ comments, the final Slovenian EN24 questionnaire version was created and presented to the EORTC Translational Office.

### 2.3. Questionnaire Evaluation

Questionnaire evaluation followed the EORTC scoring manual [19,21]. Scoring was performed using a linear transformation or raw scores for each item. The items were categorized according to the EORTC scoring guide and calculated on a scale from a minimum of 0 to a maximum of 100. Items were categorized on a functional scale reporting sexual interest (SXI), sexual activity (SXA), and sexual enjoyment (SXE) on a symptom scale. The symptom scale included the assessment of lymphedema (LY; 2 items), urological symptoms (UR; 4 items), gastrointestinal symptoms (GI; 5 items), poor body image (BI; 2 items), sexual/vaginal problems (SXV; 3 items), pain in the back and pelvis (BP; 1 item), muscular pain (MP; 1 item), hair loss (HL; 1 item), and taste changes (TC; 1 item). A higher score indicated a higher level of symptom persistence or a higher functional scale performance.

### 2.4. Statistical Analysis

Descriptive statistics were reported using median values with standard deviations. Computed items were correlated using the Spearman rank correlation. Continuous data were compared using the Mann–Whitney U test. The analysis was performed using SPSS for Mac ver. 26 (IBM Corp., Armonk, NY, USA).

## 3. Results

### 3.1. Patient Characteristics

A total of 154 women with endometrial cancer were identified from an institutional registry. Questionnaires were returned via post in a pre-defined timeframe of 3 weeks. A total of 79 women returned questionnaires (response rate of 51%). The questionnaire was inadequately completed (missing answers) by 6 patients (7.6%) in the obligatory items of the assessment scale, which warranted exclusion of those records from further analysis. Cronbach’s alpha for items in the Slovenian version of the EORTC QLQ-EN24 scale was 0.72. The median age of women was 61 years (33–83). Follow-up time was 4 years (2–5). The median body mass index (BMI) (*n* = 67) was 31 (19–52). Characteristics are described in Table 1.

Patients who responded to the follow-up assessments of PROs were diagnosed mostly in FIGO stage IA (53%, *n* = 39), IB (23%, *n* = 17), II (6%, *n* = 4), III (8%, *n* = 6), and IV (8%, *n* = 6).

### 3.2. Health-Related Symptoms

**Figure 1:**
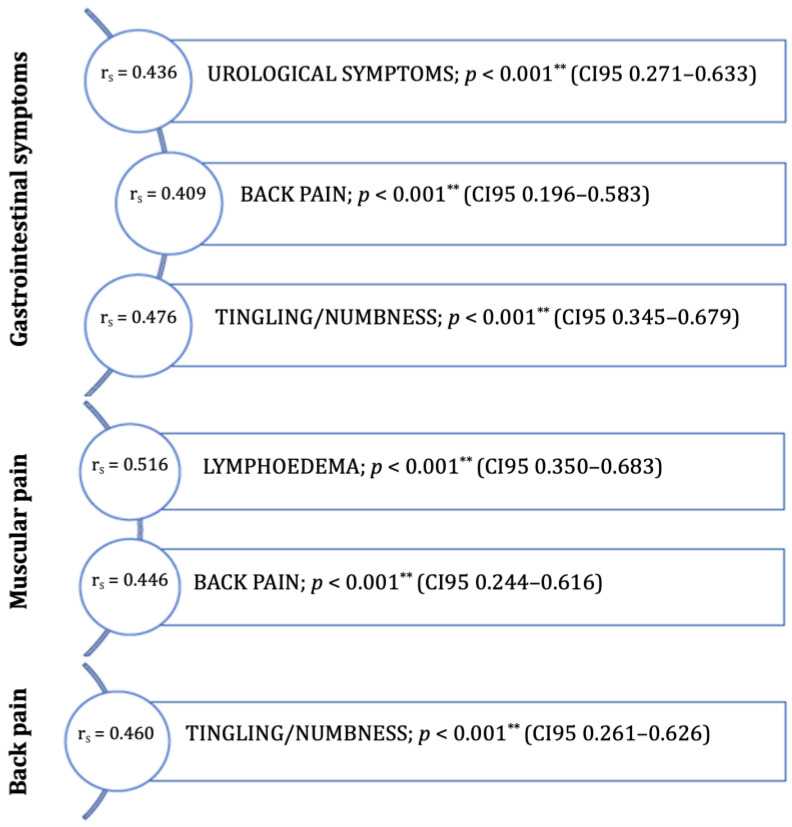
Moderate symptom correlations (r_s_ = 0.40–0.69) in women (post-treatment of endometrial cancer). ** *p* < 0.001.

All health-related symptoms are presented in Supplemental Table S1. There were several mild correlations between the reported symptoms. There were no relevant strong correlations between the health-related symptoms. Moderate correlations were reported in the correlation between GI symptoms and urological symptoms, back pain, or tingling/numbness (Figure 1). Patients reporting muscular pain shwed moderate correlations with lymphedema and back pain (Figure 1). Reporting on back pain was also significantly correlated with reports of tingling/numbness (Figure 1).

A total of 68 women were treated with surgical therapy (93%). Out of these, 45 had minimally invasive surgery (66.2%) and 23 women had open surgery (33.8%). Somatic symptoms mostly did not correlate with any surgical management characteristics. Women treated with minimally invasive surgery reported significantly worse changes in taste (*p* < 0.031). All data on the impacts of surgical treatment on the reported symptoms are depicted in Supplemental Table S2. Lymph-node treatment was performed using sentinel node biopsy (SNB; *n* = 27; 39.7%), pelvic lymphadenectomy (LND; *n* = 22; 32.4%) or SNB with contralateral pelvic LND (*n* = 7; 10.3%). There were no lymph node procedures in 12 women (17.6%). Four women received only systemic treatments and were not included in the analysis of surgical approaches and PROs. There were no significant associations between the reported symptoms and pelvic lymph node treatment (Supplemental Table S3). However, women who underwent paraaortic lymph node dissections (*n* = 6) reported more frequent gastrointestinal symptoms (*p* < 0.025) and back pain (*p* < 0.010).

Data on systemic therapy were available from the electronic records for 69 women (94.5%). After surgical treatment, 38 women had no additional adjuvant therapy (55%). A total of 18 women had adjuvant radiotherapy (26%), 4 women had adjuvant chemotherapy (5.8%), and 9 women (13%) had combined systemic therapy. Two women had neoadjuvant therapy and were added to the combined systemic treatment group, as after surgical treatment, they also had radiotherapy. There were no statistically significant differences in PROs in regard to systemic therapy (Table 2).

### 3.3. Sexual Functioning and Sexual Health-Related Symptoms

**Figure 2:**
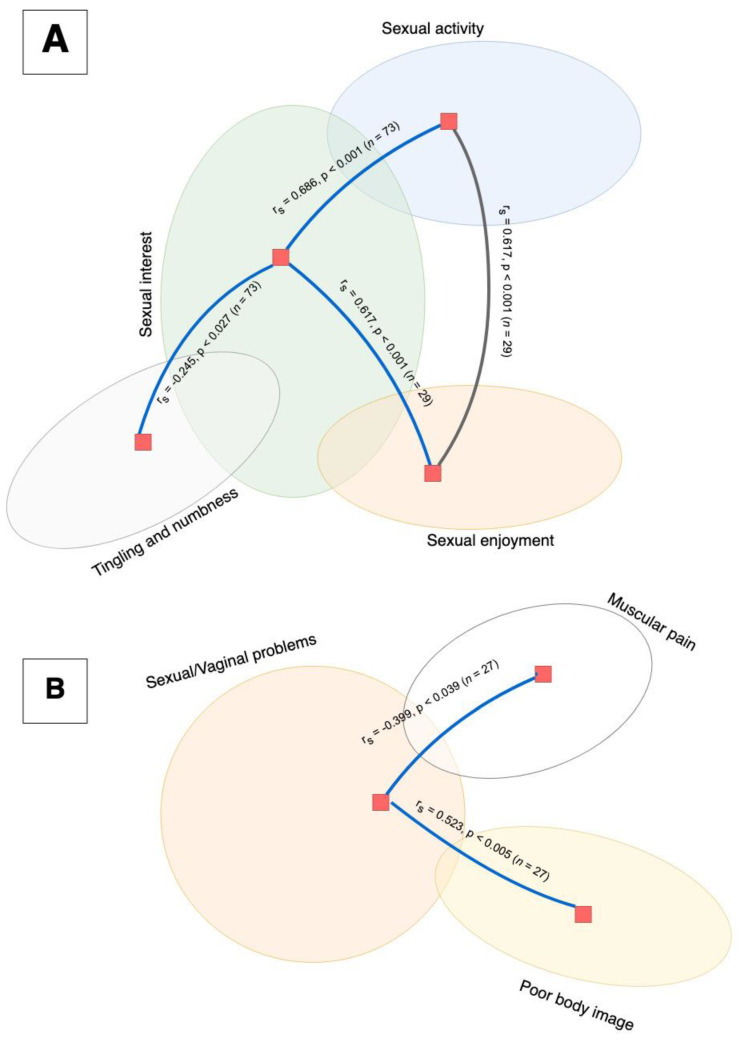
A correlation analysis using Spearman’s Rho showed that the function scale items (depicted in part (**A**)) were positively correlated. High reported sexual interest was significantly correlated to sexual activity (*p* < 0.001) and sexual enjoyment (*p* < 0.001), and was negatively correlated with tingling and numbness symptoms (*p* < 0.027). Additionally, there was a significant correlation between sexual enjoyment and activity (*p* < 0.001). None of the functional items were correlated with the symptoms of sexual/vaginal problems (depicted in part (**B**)). Sexual/vaginal problems were correlated with poor body image (*p* < 0.005) and negatively correlated with muscular pain (*p* < 0.026).

Twenty-nine women (39%) reported sexual activity within 4 weeks prior to completing the questionnaire. None of the reported symptoms, except urological (*p* > 0.021) symptoms, were significantly correlated with recent sexual intercourse (Figure 2).

Sexual health-related symptoms were not connected to somatic health symptoms, except muscular pain. In sexually active women, sexual/vaginal problems were correlated with poor body image and muscular pain but were not connected to the categories of sexual functioning. Sexual functioning was also mildly correlated with tingling and numbness.

Women in our study showed moderate correlations between sexual interest and sexual activity as well as sexual activity and sexual enjoyment.

## 4. Discussion

This study reported on the PROs using the first Slovenian translation of the EORTC QLQ-EN24 assessment scale. Our study did not show significant differences in most PROs regarding surgical and systemic therapy. The study highlighted an important need to further explore and assist in alleviating sexual health-related symptoms. Women in our study reported a significant interconnection of sexual/vaginal problems with poor body image and muscular pain, allowing us to also explore somatic assistance in improving PROs in women with endometrial cancer.

A recent study assessing quality of life across different studies has shown that outcomes vary extensively among different gynecological cancers. Thus, impacts should be assessed individually and counseling (regarding long-term data) should be performed on cancer-specific data [22]. This highlights the importance of data evaluation on PROs to assist in appropriate counseling.

Our median follow-up was 4 years. A longitudinal study on patient-reported outcomes with the use of the EORTC QLQ-EN24 symptom scale showed that symptoms of lymphedema, gastrointestinal symptoms, and lower back pain were reported as significantly worse in the first 3 months after radiotherapy treatment, but not in the 3- to 12-month period [23].

Previous reports on somatic symptoms showed that women usually develop lymphedema within 12 months of primary treatment [24]. A national Korean survey that evaluated patients between 2004 and 2017 reported a 13.1% rate of lymphedema development. Factors influencing lymphedema development were older age and multimodal treatment [25]. However, the development of lymphedema was not connected to any treatment or demographic factors reported recently. A higher level of trouble with lymphedema reported in our study was moderately connected with muscular pain.

Forty percent of gynecological cancer survivors reported previous gastrointestinal symptoms [26]. Gastrointestinal symptoms after systemic therapy for pelvic cancers are common, with some reports stating that up to 90% of patients have permanent changes in bowel movements [27]. Patients in our study reported more frequently gastrointestinal symptoms in correlation with urological symptoms, back pain, tingling, and numbness. These data point to possible correlations with therapy that were not present in our study.

There were significant changes in taste reported in our study. This might be due to the study being conducted during the COVID-19 pandemic in April 2021 and we did not adjust to study for previous COVID-19 infections. COVID-19 was previously shown to impact smell and taste [28]. Change in taste was reported as an impact of chemotherapy [29]; however, in our group, neither chemotherapy nor chemo- and radiotherapy resulted in significant changes in taste. Oddly, taste changed significantly in the minimally invasive surgery group, which could have accounted for the pandemic setting. Other reports have shown a correlation between albumin levels and taste change [30], which we did not account for in our study. Further analysis, while not statistically significant, showed that hair loss was higher in the radiotherapy group compared to the chemotherapy group (although it could have been due to other reasons rather than the therapy itself). As women undergoing chemotherapy were prepared for such effects, the level of reported troubles might have been lower than in the radiotherapy group, where sporadic hair loss might be seen as an important long-term symptom post-treatment, although it is somatically not interconnected.

Sexual functioning after primary treatment of endometrial cancer can importantly impact the quality of life in women with gynecological cancer [31]. Sexual functioning was not dependent on age or time of diagnosis in our study. A previous study showed that sexual functioning in women with endometrial cancer deteriorated, regardless of age. In particular, desire for sexual activity in elderly women after primary endometrial cancer treatment, as well as arousal and reported orgasm levels, were significantly decreased in comparison to younger women [32]. Previous meta-analyses showed that physical activity interventions were associated with sexual interest but not sexual function [6]. In a recent report on the validation of a sexual health scale, women identified sexual concerns after gynecological cancer treatment, both in the group of women who were sexually active as well as sexually inactive due to illness or treatment-associated sexual difficulties [33].

Recent data (e.g., in a cross-cancer study) showed that patient-reported outcomes could be impacted by sleep regularity. Good sleep patterns were associated with increased global QOL as well as better physical functioning. Patients with regular sleep patterns also reported less nausea/vomiting, dyspnea, and diarrhea [34]. Further studies should incorporate sleep regularity as a factor analysis.

While our study had a relatively high response rate, the results should be interpreted with caution as the group of EC survivors was relatively small. This meant that specific regimens of chemotherapy treatments could not be compared between each other (e.g., deviations from standard therapy) as these were too-small groups to statically analyze adequately. Additional assessments for more focused evaluations of sexual functioning as well as overall clinical status and comorbidities in further trials could improve the understanding of endometrial cancer. Furthermore, the EORTC QLQ-EN24 is not the only assessment scale and should also be tested against other PRO scales.

## 5. Conclusions

The Slovenian translation of the EORTC QLQ-EN24 showed adequate validity and reliability. Data showed the importance of counseling women with endometrial cancer about the possible impacts of treatment on long-term PROs. This is especially important as certain interventions in the post-primary treatment environment can improve symptoms. Our data indicate that, especially in terms of assistance with sexual or vaginal problems, more quality evidence on alleviating symptoms with pharmacological or non-pharmacological symptoms should be examined. Individualizing assistance in the post-primary treatment setting is essential in improving different aspects of PROs in women with EC.

## Figures and Tables

**Table 1:** Basic patient characteristics.

	Median Reported Values (SD) *	Age at Time of Diagnosis ^†^	Body Mass Index ^‡^
Lymphedema (*n* = 69)	34.0 (27.2)	r_s_ = 0.136, *p* < 0.252	r_s_ = 0.246, *p* < 0.045
Urological (*n* = 69)	25.0 (26.2)	r_s_ = 0.097, *p* < 0.415	r_s_ = 0.246, *p* < 0.044
GI (*n* = 69)	20.0 (17.1)	r_s_ = −0.062, *p* < 0.600	r_s_ = 0.071, *p* < 0.570
Poor body (*n* = 69)	0.00 (21.8)	r_s_ = −0.350, *p* < 0.002	r_s_ = −0.125, *p* < 0.312
Back pain (*n* = 69)	32.9 (32.9)	r_s_ = 0.161, *p* < 0.174	r_s_ = 0.077, *p* < 0.536
Tingling and numbness (*n* = 69)	31.5 (31.5)	r_s_ = 0.170, *p* < 0.150	r_s_ = 0.017, *p* < 0.890
Muscular pain (*n* = 69)	31.0 (31.0)	r_s_ = 0.149, *p* < 0.209	r_s_ = 0.211, *p* < 0.087
Hair loss (*n* = 69)	28.0 (28.0)	r_s_ = −0.037, *p* < 0.758	r_s_ = −0.007, *p* < 0.958
Taste change (*n* = 69)	28.0 (28.0)	r_s_ = 0.018, *p* < 0.878	r_s_ = −0.050, *p* < 0.687
Sexual interest (*n* = 69)	0.00 (21.1)	r_s_ = −0.408, *p* < 0.001	r_s_ = −0.103, *p* < 0.405
Sexual activity (*n* = 69)	0.00 (21.1)	r_s_ = −0.506, *p* < 0.001	r_s_ = −0.011, *p* < 0.927
Sexual enjoyment (*n* = 26)	67.0 (28.9)	r_s_ = −0.358, *p* < 0.056	r_s_ = 0.143, *p* < 0.486
Sexual/vaginal problems (*n* = 26)	12 (23.8)	r_s_ = −0.208, *p* < 0.279	r_s_ = −0.225, *p* < 0.269

* Values are reported on a scale between 0 and 100 based on an evaluation key; ^†^ continuous data (ages ranging from 33 to 83) were correlated with reported scale values; ^‡^ continuous data (BMI ranging from 19 to 52) were correlated with reported scale values.

**Table 2:** Impact of systemic therapy on patient-reported outcomes.

	No Adjuvant Therapy * (*n* = 38) (Mean, SD)	Radiotherapy * (*n* = 18) (Mean, SD)	Chemotherapy * (*n* = 4) (Mean, SD)	Radiotherapy and Chemotherapy * (*n* = 9) (Mean, SD)	Significance
Lymphedema	31 (29.8)	26.2 (23.8)	42 (32.0)	31.8 (21.1)	*p* > 0.754
Urological symptoms	29.7 (24.7)	34.1 (31.8)	25.3 (24.8)	35.6 (28.3)	*p* > 0.963
Gastrointestinal symptoms	20.7 (15.7)	23.2 (22.1)	22.0 (18.4)	21.0 (13.6)	*p* > 0.988
Poor body image	12.4 (24.6)	11.2 (20.8)	21.0 (25.1)	7.6 (12.4)	*p* > 0.787
Back pain	49.5 (31.7)	44.8 (32.3)	58.5 (42.0)	48.4 (33.8)	*p* > 0.817
Tingling and numbness	31.8 (34.7)	35.6 (26.8)	33.5 (38.7)	44.9 (28.7)	*p* > 0.611
Muscular pain	37.2 (32.7)	35.5 (33.3)	33.5 (38.7)	48.7 (17.4)	*p* > 0.594
Hair loss	20.4 (29.9)	9.4 (15.7)	8.5 (17.0)	29.8 (42.3)	*p* > 0.542
Taste change	20.4 (29.9)	7.6 (14.5)	0 (.00)	29.8 (42.3)	*p* > 0.183
Sexual interest	15.1 (21.7)	17.0 (17.5)	17.0 (19.6)	18.8 (24.5)	*p* > 0.912
Sexual activity	14.2 (21.6)	11.3 (16.5)	8.5 (17.0)	15.0 (24.4)	*p* > 0.970
Sexual enjoyment	46.2 (29.5); (*n* = 16)	56.0 (17.0); (*n* = 6)	56.0; (*n* = 1)	40.2 (43.5); (*n* = 5)	*p* > 0.750
Sexual/vaginal problems	22.8 (22.6); (*n* = 16)	37.5 (26.9); (*n* = 6)	34.0; (*n* = 1)	7.0 (10.3); (*n* = 5)	*p* > 0.085

* Values are reported on a scale between 0 and 100 based on an evaluation key and analyzed according to systemic data subtypes.

## Data Availability

Data are available upon reasonable request to the corresponding author.

## Supplementary Materials

The following supporting information can be downloaded at: www.mdpi.com/article/10.3390/curroncol29080414/s1, Table S1: Correlational matrix of symptoms., Table S2: Impact of surgical therapy on Patient-Reported Outcomes, Table S3: Impact of lymph node treatment on Patient-Reported Outcomes.
