# Supplementary material for: Evaluation of Patient-Reported Symptoms and Functioning after Treatment for Endometrial Cancer
Source: Curr Oncol. 2022 Jul 22;29(8):5213–22. doi: 10.3390/curroncol29080414 (PMC9394308; doi:10.3390/curroncol29080414)
Supplement: Supplementary file 1 [file curroncol-29-00414-s001.zip › curroncol-1747543-supplementary.pdf]

Supplementary Material

Evaluation of Patient Reported Symptoms and Functioning after Treatment for Endometrial Cancer

Monika Sobočan [1,2,*], Dorotea Gašpar [2], Estera Gjuras [2] and Jure Knez [1,2]

[1] Division of Gynecology and Perinatology, University Medical Centre Maribor, 2000 Maribor, Slovenia
[2] Department of Obstetrics and Gynecology, Faculty of Medicine, University of Maribor, 2000 Maribor, Slovenia
* Correspondence: monika.sobocan@gmail.com

Table S1. Correlational matrix of symptoms.

	**Lymph-edema**	**Urological**	**GI**	**PoorBody**	**BackPain**	**Tingling and numbness**	**Muscular pain**	**Hair loss**	**Taste change**
**Lymph-edema**	N/A	r_s = 0.374, *p* < 0.001** (CI95 0.164-.561)	r_s = 0.226, *p* = *0*.055 (CI95 0.080-.500)	r_s = 0.095, *p* = *0*.423 (CI95 -.097-.355)	rs = 0.293, *p* = *0*.012* (CI95 0.062-.486)	rs = 0.490, *p* < *0*.001** (CI95 0.264-.628)	rs = 0.516, *p* < *0*.001** (CI95 0.350-.683)	rs = 0.076, *p* = *0*.523 (CI95 -.183-.276)	rs = 0.101, *p* < *0*.394 (CI95 -.166-.292)
**Urological**	rs = 0.374, *p* < 0.001** (CI95 0.164-0.561)	N/A	rs = 0.436, *p* < 0.001** (CI95 0.271-.633)	rs = 0.145, *p* = *0*.220 (CI95 0.019-.453)	rs = 0.251, *p* = *0*.032* (CI95 0.002-.439)	rs = 0.376, *p* < *0*.001** (CI95 0.197-.584)	rs = 0.270, *p* = *0*.021* (CI95 0.034-.464)	rs = 0.322, *p* = *0*.005** (CI95 0.067-.490)	rs = 0.351, *p* = *0*.002** (CI95 0.088-.506)
**GI**	rs = 0.226, *p* = *0*.055 (CI95 0.080-.500)	rs = 0.436, *p* < *0*.001** (CI95 0.271-.633)	N/A	rs = 0.133, *p* = *0*.264 (CI95 0.036-.466)	rs = 0.409, *p* < *0*.001** (CI95 0.196-.583)	rs = 0.476, *p* < *0*.001** (CI95 0.345-.679)	rs = 0.213, *p* = *0*.070 (CI95 0.030-.461)	rs = 0.126, *p* = *0*.287 (CI95 -.133-.323)	rs = 0.176, *p* = *0*.136 (CI95 -.074-.375)
**PoorBody**	rs = 0.095, *p* = *0*.423 (CI95 -.097-.355)	rs = 0.145, *p* = *0*.220 (CI95 0.019-.453)	rs = 0.133, *p* = *0*.264 (CI95 0.036-.466)	N/A	rs = 0.128, *p* = *0*.279 (CI95 -.055-.392)	rs = 0.098, *p* = *0*.409 (CI95 0.036-.466)	rs = 0.141, *p* = *0*.233 (CI95 -.120-.344)	rs = 0.059, *p* = *0*.619 (CI95 -.079-.371)	rs = 0.090, *p* = *0*.448 (CI95 -.074-.375)
**BackPain**	rs = 0.293, *p* = *0*.012* (CI95 0.062-.486)	rs = 0.251, *p* = *0*.032* (CI95 0.002-.439)	rs = 0.409, *p* < *0*.001** (CI95 0.196-.583)	rs = 0.128, *p* = *0*.279 (CI95 -.055-.392)	N/A	rs = 0.460, *p* < *0*.001** (CI95 0.261-.626)	rs = 0.446, *p* < *0*.001** (CI95 0.244-.616)	rs = 0.059, *p* = *0*.618 (CI95 -.161-.297)	rs = 0.141, *p* = *0*.233 (CI95 -.111-.343)
**Tingling and numbness**	rs = 0.490, *p* < *0*.001** (CI95 0.264-.628)	rs = 0.376, *p* < *0*.001** (CI95 0.197-.584)	rs = 0.476, *p* < *0*.001** (CI95 0.345-.679)	rs = 0.098, *p* = *0*.409 (CI95 0.036-.466)	rs = 0.460, *p* < *0*.001** (CI95 0.261-.626)	N/A	rs = 0.387, *p* < *0*.001** (CI95 0.198-.584)	rs = 0.160, *p* = 176 (CI95 0.034-.464)	rs = 0.262, *p* = *0*.025* (CI95 0.113-.524)
**Muscular pain**	rs = 0.516, *p* < *0*.001** (CI95 0.350-.683)	rs = 0.270, *p* = *0*.021* (CI95 0.034-.464)	rs = 0.213, *p* = *0*.070 (CI95 0.030-.461)	rs = 0.141, *p* = *0*.233 (CI95 -.120-.334)	rs = 0.446, *p* < *0*.001** (CI95 0.244-.616)	rs = 0.387, *p* < *0*.001** (CI95 0.198-.584)	N/A	rs = 0.097, *p* = *0*.413 (CI95 -.117-.337)	rs = 0.161, *p* = *0*.172 (CI95 -.076-.373)
**Hair loss**	rs = 0.076, *p* = *0*.523 (CI95 -.183-.276)	rs = 0.322, *p* = *0*.005** (CI95 0.067-.490)	rs = 0.126, *p* = *0*.287 (CI95 -.133-.323)	rs = 0.059, *p* = *0*.619 (CI95 -.079-.371)	rs = 0.059, *p* = *0*.618 (CI95 -.161-.297)	rs = 0.160, *p* = 176 (CI95 0.034-.464)	rs = 0.097, *p* = *0*.413 (CI95 -.117-.337)	N/A	rs = 0.907, *p* < *0*.001** (CI95 0.936-.974)
**Taste change**	rs = 0.101, *p* < *0*.394 (CI95 -.166-.292)	rs = 0.351, *p* = *0*.002** (CI95 0.088-.506)	rs = 0.176, *p* = *0*.136 (CI95 -.074-.375)	rs = 0.090, *p* = *0*.448 (CI95 -.074-.375)	rs = 0.141, *p* = *0*.233 (CI95 -.111-.343)	rs = 0.262, *p* = *0*.025* (CI95 0.113-.524)	rs = 0.161, *p* = *0*.172 (CI95 -.076-.373)	rs = 0.907, *p* < *0*.001** (CI95 0.936-.974)	N/A

Table S2. Impact of surgical therapy on Patient Reported Outcomes

	**Minimally invasive therapy (Mean, SD)**	**Open surgery (Mean, SD)**	**Significance**
Lymphoedema	30.0 (26.2)	38.7 (28.7)	$p > 0.221$
Urological	31.6 (26.8)	32.6 (26.8)	*p*. > 855
Gastrointestinal	21.6 (16.7)	23.4 (18.7)	*p*. > 881
Poor Body image	12.0 (22.2)	11.7 (22.9)	*p*. > 0.999
Back Pain	47.8 (30.0)	56.8 (35.4)	$p > 0.249$
Tingling and numbness	37.4 (32.0)	33.6 (31.9)	$p > 0.688$
Muscular pain	34.4 (30.0)	46.7 (32.9)	$p > 0.126$
Hair loss	20.2 (28.0)	13.1 (29.8)	$p > 0.133$
Taste change	20.2 (28.0)	10.2 (29.2)	$p > 0.031$
Sexual interest	14.3 (18.4)	16.2 (22.4)	$p > 0.892$
Sexual activity	12.8 (19.4)	11.8 (19.3)	$p > 0.818$
Sexual enjoyment	44.2 (27.4); ($n = 19$)	46.3 (30.5); ($n = 8$)	$p > 0.955$
Sexual/Vaginal problems	23.9 (25.4); ($n = 19$)	26.9 (22.3); ($n = 8$)	$p > 0.607$

Table S3. Impact of lymph node treatment on Patient Reported Outcomes

	**SNB (Mean, SD)**	**LND (Mean, SD)**	**SNB with LND (Mean, SD)**	**no LN treatment (Mean, SD)**	**Significance**
Lymphoedema	28.1 (25.9)	39.7 (28.9)	26.6 (21.3)	35.1 (29.9)	$p > 0.488$
Urological	28.4 (23.8)	36.0 (27.3)	26.4 (29.2)	35.8 (31.4)	$p > 0.734$
Gastrointestinal	21.1 (14.3)	20.9 (18.7)	24.9 (13.1)	25.8 (23.5)	$p > 0.830$
Poor Body image	11.3 (22.4)	9.2 (17.8)	9.6 (18.9)	19.6 (31.0)	$p > 0.738$
Back Pain	48.5 (28.2)	60.9 (31.8)	33.9 (19.3)	47.4 (41.3)	$p > 0.191$
Tingling and numbness	30.0 (28.4)	36.7 (30.8)	52.9 (26.0)	39.1 (42.2)	$p > 0.374$
Muscular pain	37.4 (29.8)	42.7 (36.0)	33.9 (19.3)	36.4 (33.3)	$p > 0.924$
Hair loss	15.0 (21.6)	12.2 (26.4)	19.1 (37.8)	33.6 (37.6)	$p > 0.204$
Taste change	13.8 (21.4)	10.7 (26.1)	19.1 (37.8)	33.6 (37.6)	$p > 0.126$
Sexual interest	13.9 (17.0)	16.9 (22.7)	9.7 (16.6)	16.9 (22.7)	$p > 0.902$
Sexual activity	15.1 (19.5)	10.8 (19.2)	0 (.00)	16.9 (22.7)	$p > 0.192$
Sexual enjoyment	44.8 (29.7); ($n = 12$)	52.7 (32.5); ($n = 7$)	0; ($n = 1$)	43.4 (16.1); ($n = 7$)	$p > 0.417$
Sexual/Vaginal problems	19.2 (16.7); ($n = 12$)	30.6 (25.6); ($n = 7$)	0; ($n = 1$)	32.1 (33.0); ($n = 7$)	$p > 0.451$

LN: lymph-node, SNB: sentinel lymph-node biopsy, LND: lymphadenectomy, SNB with LND: SNB with contralateral LND.
